# Secondary Full-thickness Macular Holes after Diabetic Vitrectomy: Clinical Manifestations and Rational Approaches to the Treatment

**DOI:** 10.1155/2022/3156642

**Published:** 2022-05-31

**Authors:** Yun Hsia, Chung-May Yang, Yi-Ting Hsieh, Lu-Chun Wang

**Affiliations:** ^1^National Taiwan University Hospital Jin-Shan Branch, New Taipei City, Taiwan; ^2^Department of Ophthalmology, National Taiwan University Hospital, Taipei, Taiwan; ^3^College of Medicine, National Taiwan University, Taipei, Taiwan; ^4^Department of Ophthalmology, National Taiwan University Hospital Yunlin Branch, Douliu, Yunlin County, Taiwan

## Abstract

**Purpose:**

The aim of the study is to present the clinical characteristics and surgical treatment of secondary full-thickness macular hole (MH) after diabetic vitrectomy (DV) in patients with proliferative diabetic retinopathy (PDR).

**Methods:**

In this retrospective, observational, and longitudinal study, we enrolled consecutive patients with PDR who developed MH after DV. The macular structure was evaluated using optical coherence tomography. The clinical characteristics, surgical techniques, and outcomes were also recorded.

**Results:**

Three patients developed MH within 6 weeks, which was associated with foveal thinning, residual fibrovascular proliferation, or anterior proliferative vitreoretinopathy. Six patients developed MH originating from the epiretinal membrane (ERM) with lamellar MH (LMH) after a median interval of 16.5 months. Three of them were complicated with retinal detachment (RD). Various surgical procedures were performed according to the clinical scenarios, including internal limiting membrane (ILM) peeling, inverted ILM flap insertion, temporal inverted ILM flap, lens posterior capsular flap insertion, and neurosensory retinal free flap insertion. All patients achieved MH closure after surgery, and 5 patients exhibited improved visual acuity.

**Conclusions:**

MH may develop after successful DV, with a high rate of associated RD. Rapid MH formation was attributed to unreleased tractional force and weakened foveal structure. The development of ERM and LMH also led to MH. Various surgical techniques could be used for MH closure.

## 1. Introduction

Full-thickness macular hole (MH) may develop in eyes with proliferative diabetic retinopathy (PDR) via distinct pathways [1] and has a prevalence of approximately 1.2% [2, 3]. Previous studies have elaborated on the clinical manifestations and management strategies of MH in non-operated eyes with PDR [1–9]. However, MH may develop after diabetic vitrectomy (DV) in patients with PDR.

Secondary MH after pars plana vitrectomy (PPV) has been extensively studied in cases of retinal detachment (RD) [10–12], epiretinal membrane (ERM) [13], and myopic traction maculopathy [14, 15]. The most common predisposing factors include ERM formation, internal limiting membrane (ILM) traction, weakened foveal structure, and vitreoschisis [11]. Secondary MHs may have distinct pathogenesis and clinical manifestations depending on the underlying disease. However, to our knowledge, no studies have discussed secondary full-thickness MH after DV in detail.

Owing to the specific microenvironment and abnormal macular structure after DV, post-DV MH might exhibit clinical features different from those of other postoperative MHs. Additionally, the management of MH after DV may be complicated by weakened retinal structure and adherent residual fibrotic tissue. The conventional ILM peeling technique may not be accessible or suitable, and various recently developed surgical techniques may be required to suit different macular conditions [9, 16–26]. Depending on the macular condition, a tailored technique may be needed to obtain the best anatomical results.

This study investigated various clinical scenarios associated with secondary MH after DV and explored the appropriate surgical treatment to achieve MH closure.

## 2. Materials and Methods

The clinical charts of all patients with PDR who received DV between January 2010 and November 2020 at the National Taiwan University Hospital were retrospectively reviewed. All data collection and analyses were conducted in accordance with the tenets of the Declaration of Helsinki. Patients who developed secondary MH after primary DV were enrolled, while those with a history of PPV for other diseases or persistent MH from the primary DV were excluded from this study. The study was approved by the institutional review board of the National Taiwan University Hospital. The requirement for informed consent was waived owing to the retrospective nature of the study.

All patients underwent an initial vitrectomy for the treatment of PDR complications, including vitreous hemorrhage (VH), fibrovascular proliferation (FVP), or tractional retinal detachment (TRD). Patient demographic data, indications for DV, findings of dilated fundus examination, best-corrected visual acuity (BCVA) before and after each operation, the interval between the last DV and MH development, surgical procedures, and MH repair techniques were documented. The extent of FVP was classified into four grades according to the definition of a previous study [27]. FVP activity was classified as “active” (if neovascularized tissue or any degree of VH was identified) or “mainly fibrosis.” [27] The macular structure was evaluated using optical coherence tomography (OCT) (Stratus OCT, Carl Zeiss Meditec, Inc., Dublin, CA, USA; Cirrus OCT, Carl Zeiss Meditec, Inc., Dublin, CA, USA; or RTVue Premier, Optovue, Inc., Fremont, CA, USA). The presence of ERM and cystoid macular edema (CME) was recorded. A manual caliper was used to measure MH size. All patients underwent follow-ups for >3 months after the final surgical procedure.

### 2.1. Surgical Techniques

For MH repair surgery, two retinal specialists (C. M. Y. and Y. T. H.) performed standard three-port 23- or 25-gauge PPV. The fibrovascular membranes were removed using scissors, forceps, and vitrectomy probes. Indocyanine green (ICG)-assisted ILM peeling was performed in cases without adequate ILM removal from previous surgeries. Different surgical techniques have been used for the treatment of MH, including standard ILM peeling, inverted ILM flap technique, temporal inverted ILM flap technique, neurosensory retinal flap, and lens anterior or posterior capsular flap insertion into the MH. The indications for different surgical techniques were as follows. Standard ILM peeling was performed if no ILM peeling had been performed in the previous surgery and if the MH was < 500 *μ*m in diameter. Temporal inverted ILM flap or ILM flap insertion was performed if the MH was > 500 *μ*m in diameter or if RD was observed. Lens anterior capsular flap insertion was performed if cataract surgery was performed in the same setting, without available ILM tissue. Lens posterior capsular flap insertion was performed in pseudophakic eyes without available ILM tissues. A neurosensory retinal flap was created in eyes with complex RD lacking adequate ILM or lens capsular tissue. After the essential procedures were performed, simple air-fluid exchange or perfluorocarbon liquid injection followed by air-fluid exchange was performed depending on the retinal status. The vitreous cavity was flushed with 15% perfluoropropane (C_3_F_8_) or silicone oil (SO).

## 3. Results

This study enrolled 9 eyes from 9 patients (4 male and 5 female). The average patient age was 57.2 ± 6.1 years. The time interval between the last DV and MH formation varied significantly. Three patients (Cases 1–3) developed MH rapidly (at 2, 3, and 5 weeks, respectively). Cases 1 and 2 showed decreased minimal foveal thickness (135 and 87 *μ*m, respectively) after SO removal. Case 2 also had an extensive and thickened ERM at the arcades, which was exerting tractional force on the macular area. Case 3 had severe anteroposterior traction due to anterior proliferative vitreoretinopathy (PVR). Cases 2 and 3 had macular hole retinal detachment (MHRD). Five patients (Cases 4–9) developed MH > 6 months after DV (median: 16.5 months, range: 6.5–76 months). All patients developed ERM during the follow-up, and 4 patients developed lamellar MH (LMH) before the development of full-thickness MH. Only one of these patients had undergone ILM peeling during the initial DV. All patients with ERM had active and grade 2 or higher FVP. The median interval to ERM formation after DV was 7 months (range: 1–26 months). The median interval from ERM growth to MH formation was 12 months (range: 3–50 months). Case 8 had chronic RD and underwent multiple surgeries.

Regarding MH repair surgeries, Case 1 underwent lens posterior capsular flap insertion. Cases 2 and 3 had MHRD and underwent inverted ILM flap and neurosensory retinal free flap insertion, respectively. Cases 4–7 and 9 underwent ERM removal and ILM peeling, while Cases 7 and 9 underwent an additional temporal inverted ILM flap technique. Case 8 underwent ILM peeling, subfoveal band removal, and laser supplementation at the MH margin. All cases achieved MH closure.  Table 1 summarizes the clinical data of all patients. The following sections briefly describe 4 representative cases.

### 3.1. Case 1

**Figure 1:**
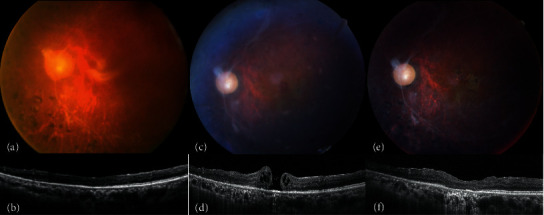
(a, b) A patient is filled with silicone oil (SO) after a previous diabetic vitrectomy. (c, d) Two weeks after receiving SO removal and cataract operation, a full-thickness macular hole (MH) develops. (e, f) Lens posterior capsular flap insertion and intravitreal infusion of C_3_F_8_ successfully seal the MH.

A 54-year-old man with PDR and TRD underwent PPV and intravitreal SO injection at another hospital. Two years later, he visited our hospital with a BCVA of 20/100. He underwent cataract surgery, intravitreal SO removal, ICG-assisted ILM peeling, and residual FVP removal. Macular thinning was noted after the surgery. MH developed 2 weeks later, and his BCVA decreased to 20/400. Lens posterior capsular flap insertion and intravitreal infusion of C_3_F_8_ were then performed. The MH was sealed, and his BCVA had improved to 20/125 at 13 months (Figure 1).

### 3.2. Case 2

**Figure 2:**
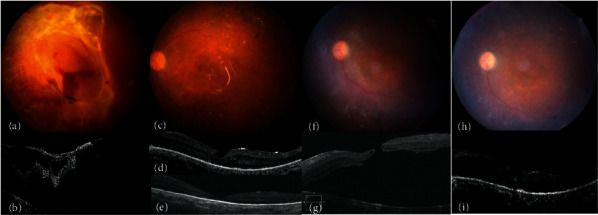
(a, b) A patient with vitreous hemorrhage, grade 3 fibrovascular proliferation, and tractional retinal detachment at presentation. (c, d) After surgery, the retina is attached under silicone oil (SO) tamponade. Optical coherence tomography shows foveal thinning and epiretinal membrane (ERM). (e) Progressive foveal thinning and the formation of lamellar macular hole (MH) are noted after SO removal. (f, g) Full-thickness MH and retinal detachment develop 3 weeks later. (h, i) The retina is attached and the MH is sealed after ERM removal, internal limiting membrane peeling, and inverted internal limiting membrane flap insertion.

A 50-year-old woman presented with PDR, grade 3 FVP, and TRD. She underwent PPV and intravitreal SO infusion in the left eye. After the surgery, her BCVA improved to 20/63. Fourteen months later, her BCVA had deteriorated to 20/320, with dense cataract formation. Phacoemulsification with posterior chamber intraocular lens implantation and intravitreal SO removal were performed. However, an MH 308 *μ*m in size and recurrent RD confined to the posterior pole were noted 3 weeks after the surgery. Her BCVA was 20/200. During MH repair surgery, thickened ERMs at the superotemporal and inferotemporal arcades connected to the underlying vessels were noted. ERM removal, ICG-assisted ILM peeling, inverted ILM flap insertion into the MH, and intravitreal infusion of 15% C_3_F_8_ were performed. The MH was sealed, and her BCVA was 20/125 10 months after the surgery (Figure 2).

### 3.3. Case 3

**Figure 3:**
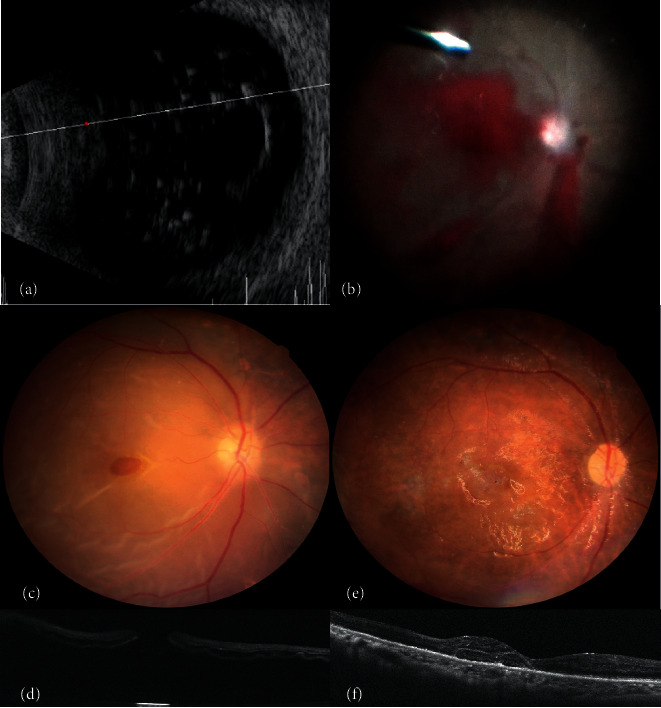
(a, b) Preoperative ultrasonography shows dense vitreous hemorrhage. Grade 3 fibrovascular proliferation and tractional retinal detachment are noted intraoperatively. (c, d) Five weeks after surgery, recurrent retinal detachment, full-thickness macular hole (MH), and temporal lower anterior proliferative vitreoretinopathy develop. (e, f) The retina is attached, and the MH is sealed after perfluorocarbon-assisted inferior 150-degree retinectomy, neurosensory retina-free flap insertion, and intravitreal silicone oil injection.

A 68-year-old woman presented with PDR complicated with VH and TRD. At presentation, her BCVA was counting fingers at 50 cm in the right eye. Therefore, she underwent PPV, FVP removal, ICG-assisted ILM peeling, and intravitreal infusion of C_3_F_8_. During surgery, peripheral temporal lower traction was noted, and iatrogenic breaks were induced. Recurrent RD and MH measuring 1063 *μ*m developed 5 weeks later, and her BCVA had deteriorated to hand motion at 1 m. Extensive anterior PVR exerting tractional force on the macular area was noted intraoperatively. Perfluorocarbon-assisted inferior 150-degree retinectomy, neurosensory retina-free flap insertion, and intravitreal SO injection were performed. The MH was sealed, and her BCVA was 20/400 1 year later (Figure 3).

### 3.4. Case 5

**Figure 4:**
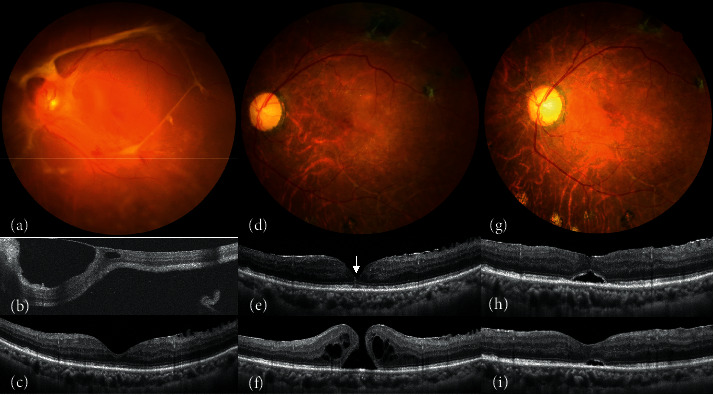
(a, b) A patient presenting with grade 3 fibrovascular proliferation, tractional retinal detachment, and rhegmatogenous retinal detachment. (c) Six months after surgery, optical coherence tomography (OCT) shows relatively normal foveal contour along with epiretinal membrane (ERM) on the temporal side. (e) The ERM becomes more significant, and lamellar macular hole (MH) develops during follow-up. Foveal-crack sign, a vertical hyperreflective line, is demonstrated by OCT (arrow). (d, f) A full-thickness MH develops 5 months later. (g-i) After ERM removal and ILM peeling, the MH has been sealed with gradual reabsorption of the subretinal fluid.

A 54-year-old woman had PDR complicated by grade 3 FVP, TRD, and rhegmatogenous RD (RRD). Her initial BCVA was hand motion at 20 cm. She underwent PPV, triamcinolone and ICG-assisted ILM peeling, FVP removal, and intravitreal infusion. After surgery, the retina was reattached and her BCVA improved to 20/125. During follow-up, ERM and LMH were noted. The LMH progressed to a full-thickness MH 11 months after the initial surgery and her BCVA deteriorated to 20/200. She underwent ERM removal, ICG-assisted ILM peeling, and intravitreal C_3_F_8_ infusion. The MH was sealed, and her final BCVA was 20/100 6 months later (Figure 4).

**Figure 5:**
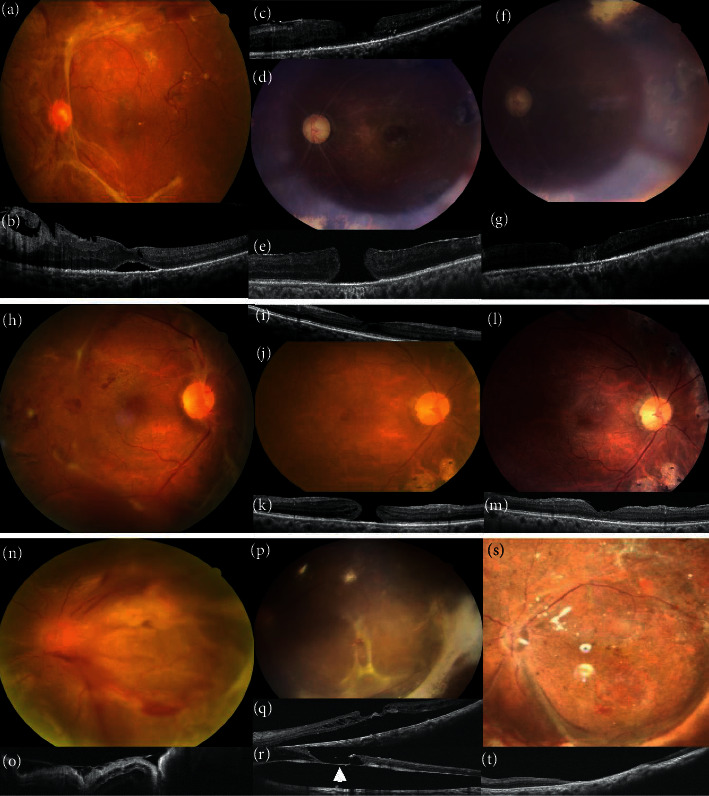
(a-g) A patient (Case 6) has (a, b) grade 2 fibrovascular proliferation (FVP) and tractional retinal detachment (TRD). (c) Eight months after surgery, optical coherence tomography (OCT) shows lamellar macular hole (MH) and epiretinal membrane (ERM). (d, e) A 750-*μ*m MH develops 21 months after cataract operation and silicone oil removal. (f, g) The patient receives ERM removal, internal limiting membrane (ILM) peeling, and temporal inverted ILM flap. The MH is sealed postoperatively. (h-m) A patient (Case 7) who presented with (h) grade 3 FVP and vitreous hemorrhage (VH). (i-k) Lamellar MH and ERM are noted and progress into full-thickness MH at 76 months after the initial surgery. (l, m) After the cataract operation, ERM removal, ILM peeling, and inverted temporal ILM flap, the MH is sealed. (n-t) The patient (Case 8) who presented with (n, o) grade 4 FVP, VH, TRD, and rhegmatogenous retinal detachment. (p, q) OCT shows persistent subretinal fluid and lamellar MH after multiple surgeries for recurrent RD. (r) It progresses into full-thickness MH with RD. Subretinal fibrotic bands are visible on the base of the MH intraoperatively (arrow). (s, t) The MH converts to gap-closure 1 month later.

Images of Cases 6–8 are shown in Figure 5. As the evolutions of Cases 4 and 9 were similar to those of Cases 6–8, the figures are not presented. The possible factors related to MH formation in this series are summarized in Table 2. In brief, ERM was the most common factor. Post-DV eyes with ERM had the longest time to MH formation and the lowest association with RD. On the other hand, eyes with incomplete FVP removal and PVR showed a strong association with MHRD.

## 4. Discussion

Post-vitrectomy MH has been reported after surgery for RRD, ERM, or myopic tractional maculopathy [11, 14, 28], with incidence rates ranging from 0.2% to 1.9% [14]. The possible mechanisms include iatrogenic trauma or microhole formation during the induction of posterior vitreous detachment or membrane peeling, and CME tangential traction caused by vitreoschisis or ERM, and the underlying disease requiring PPV [10, 11, 29, 30]. Secondary MH could also occur after DV for the complications of PDR. However, only a few case reports have described this non-idiopathic post-surgical condition [4, 14, 30–35]. ERM and premacular fibrosis were the most common predisposing factors for secondary MH from the limited case reports. Most patients underwent ERM removal with or without ILM peeling for MH repair. In the literature, the evolutionary process and rationale for surgical management have not yet been elaborated in detail. In the present study, we focused on the secondary MHs after DV and described their clinical characteristics, possible mechanisms, and rationale for their treatment.

The major predisposing factors for secondary MHs after DV included ERM, residual FVP, anterior PVR, chronic CME, weakened foveal structure, and iatrogenic traction during surgery. The foveal structure is weakened in eyes with PDR due to longstanding CME and ischemia [2]. Surgical manipulation during DV, induction of posterior vitreous detachment, ERM removal, and ILM peeling may further damage fragile foveal structures. Previous reports on MH secondary to RD repair surgery or DV have observed rapid MH formation due to direct traction on the macula during vitrectomy [11, 32, 34]. Case 1 developed MH 2 weeks after SO removal. Foveal thinning implied a long-standing ischemic environment and atrophy, which may increase patient susceptibility to poor visual and anatomical outcomes. Due to simultaneous ERM and ILM peeling during the SO removal surgery and the absence of a residual premacular membrane, mechanical trauma during membrane peeling may have further contributed to MH development. Moreover, residual FVP and subsequent ERM formation or even anterior PVR could lead to tangential traction in cases complicated with RD. Case 2 developed MH and localized RD 3 weeks after SO removal. During MH repair surgery, thickened ERMs connected to the retinal vessels were observed near the superotemporal and inferotemporal arcades, which caused traction on the macula. The adhesion of membranes to the vessels suggested that the membranes might represent incompletely removed FVP from the previous surgery or proliferation from the residual FVP stumps. Strong tractional force on the fovea induced MHRD. Previous case reports also demonstrated that premacular fibrotic membranes could induce rapid secondary MH formation within 2 months [33, 34]. In Case 3, recurrent RD with MH formation was observed 5 weeks after primary DV with ILM peeling. The anterior PVR in the inferior retina induced a large MH. An inferior 150-degree retinectomy was required to release traction and a neurosensory retinal flap was used to close the MH. Remote traction, if sufficiently extensive, can induce rapid MH formation. Although the strong traction from residual FVP and PVR changes induced rapid MH formation and RD, visual improvement could still be expected with prompt and proper management.

ERM was the most common predisposing factor for secondary MH after DV as reported in the literature [4, 30, 31, 35]. The tangential traction exerted on the weakened fovea caused subsequent MH formation within 7 months to 5 years [4, 30, 31, 35]. In the present study, Cases 4–9 (6 eyes) also showed ERM and subsequent MH formation after primary DV. LMH occurred in 4 eyes (67%) as a pre-MH condition. ERM develops in more than half of post-DV eyes and has a multifactorial pathogenesis [36]. The ischemic environment in eyes with PDR may persist or even worsen after DV, increasing the risk of cellular infiltration and epimacular membrane formation. Intraoperative bleeding or postoperative recurrent hemorrhage produces a pro-inflammatory environment and stimulates ERM formation, especially in patients with SO infusion [37, 38]. Eyes with active and high-grade FVPs that were more difficult to remove were prone to significant ERM formation since residual FVP or posterior hyaloid could serve as a scaffold. Meticulous ILM peeling during primary DV ensures complete removal of the preretinal membrane and vitreoschisis, and prevents ERM recurrence in post-DV eyes [39–41]. Similarly, in eyes receiving PPV for RD, not using a vitreous staining agent in the primary surgery may leave residual cortical vitreous in the premacular area, leading to ERM formation and contraction from the residual vitreous [10]. In our cohort, 77.8% of eyes (Cases 2, 4–9) showed ERM formation after DV in a median of 7 months. Six of them (85.7%) did not undergo ILM peeling during primary DV. In these patients, ERM was the main factor contributing to MH formation. Due to the relatively weak traction, MH development was delayed in these cases (median: 12.0 months) compared to patients with other contributing factors. The association with MHRD was also low (29%). In the literature, the interval from the initial surgery to secondary MH formation ranged from 1.6 to 26 months and is highly variable depending on the underlying conditions [14, 30]. Generally, eyes with myopic foveoschisis show the highest incidence and shortest interval to secondary MH formation [29]. Eyes with a history of macula-off RD, pseudohole, and CME developed secondary MH after an interval of approximately 1 year [10–12, 28, 30]. Eyes that received PPV for idiopathic ERM exhibited the most extended interval to the development of secondary MH [30]. The time to MH formation in our cohort was similar to those in eyes with macula-off RD, pseudoholes, and CME. Surprisingly, patients with ERM had limited visual improvement compared to the pre-DV status, which could be attributed to the chronicity of MH and possible delayed diagnosis.

In our cohort, one-third of the patients had MHRD. Among patients who developed MH after undergoing PPV for RD, those who underwent multiple surgeries or whose eyes were complicated with PVR exhibited a higher incidence of MHRD [10]. The complex traction and fragile structure in post-DV eyes resembled the conditions in highly myopic eyes, which also showed a higher incidence of MHRD. In our series, the predisposing factors of MH, such as incomplete FVP removal and PVR changes, had the highest associations with RD. Case 2 had foveal thinning, ERM, and subsequent LMH formation, which were worsened by residual FVP, and progressed to MHRD 3 weeks after SO removal. Case 3 had recurrent RD with concurrent MH due to severe anterior PVR 5 weeks after primary DV. Case 8 underwent multiple surgeries due to persistent chronic TRD and PVR changes. LMH developed gradually after primary DV and progressed to a full-thickness MH. When managing patients with PDR, ILM peeling during primary DV can ensure more complete removal of the premacular membrane and prevent subsequent traction. However, delicate surgical manipulation is also essential for preventing MH formation. Despite more complicated conditions, visual improvement can still be expected after successful MH repair surgery.

Strategies for managing MH in nonoperated PDR eyes have been reported [3–9]. Complete ERM removal without ILM peeling may be sufficient for eyes with moderate-to-high macular elevation [5]. The standard ILM peeling technique can successfully treat most cases and achieve better anatomical and visual outcomes [3, 8]. Traction release by the surgery and the pre-proliferative environment could help promote MH closure [3]. However, in more severe cases complicated by MHRD, other advanced techniques may be needed. An inverted epiretinal ILM flap may be preferred because it has the least interference with the foveal structure. If the ILM flap fails to cover the MH, inverted ILM flap insertion or free ILM flaps could be considered [9]. The management of secondary MH after DV has rarely been discussed in the literature. In previous case reports, most patients received primary DV due to non-clearing VH [4, 14, 31–33], which was less complicated than in our cases. Most patients underwent ERM removal with or without ILM peeling as MH repair surgery. Depending on the complicated conditions in the post-DV MHs, we applied different surgical techniques for MH repair, in which meticulous ILM and ERM peeling and removal of residual FVP were pivotal for sealing MHs and preventing recurrence. In more complicated cases, the inverted ILM flap was the first choice (Cases 7 and 9). Case 2 was treated with inverted ILM insertion instead of an inverted ILM flap because the small ILM flap tended to flip back. In eyes without available ILM tissue, lens capsular flap or neurosensory retinal flaps were utilized. Neurosensory retinal flaps are useful for complicated RD because peripheral retinectomy provides retinal tissue, drains subretinal fluid, and releases traction [9]. In Case 8, only ERM removal and ILM peeling were performed because the MH was too large for adequate ILM flap coverage or insertion. As a 360-degree retinectomy had been performed in the previous surgery, we hesitated to sacrifice any viable retinal tissue for the neurosensory flap. Gap closure of the MH was achieved. In such cases, an amniotic membrane graft may be another option [24]. Although the MH closure rate was 100% in this study, the visual prognosis was guarded in these patients due to complicated PDR and concomitant MH. The final visual acuity was poor compared to that in patients with other types of secondary MHs [10, 14, 30]. However, all patients showed better visual acuity than at the time of MH formation. Five patients exhibited visual improvement compared to the values before DV. The final visual acuity was non-inferior to eyes with PDR complicated with MH before DV [5, 19].

This study included a limited number of patients. However, the incidence of secondary MH following DV is low. To our knowledge, no previous study has focused solely on the pathogenesis and clinical features of MH secondary to DV. We observed the rapid development of MH in the presence of unreleased traction and a weakened foveal structure due to diabetic retinopathy and iatrogenic trauma. Traction from the ERM could lead to the formation of LMH, subsequently progressing to full-thickness MH. Secondary MH after DV was prone to MHRD and has a poor visual prognosis. Tailored MH repair surgery is required, considering the complicated and challenging conditions in post-DV eyes. Complete but meticulous ILM peeling is necessary to ensure the total traction release and prevent MH formation.

## Figures and Tables

**Table 1:** Clinical characteristics of the patients with secondary macular hole after diabetic vitrectomy.

	Sex/Age	Diagnosis^a^/ FVP grade/activity	Pre-op BCVA	Surgical techniques during DV	BCVA with MH	Pre-MH condition	Size/Interval of MH development (mos)	MHRD	MH surgery	MH closure;BCVA; Follow-up (mos)
1	M/54	2, 3/not available^c^	20/100	1st: SOI^*c*^ 2^nd^: SOR, ILMP	20/400	Foveal thinning, CME	313/0.5	−	Lens posterior capsular flap insertion, C_3_F_8_	+; 20/125; 48
2	F/50	1–3/3/active	20/630	1^st^: FVPR, SOI 2^nd^: SOR^b^	20/200	ERM, LMH, CME, residual FVP, foveal thinning	308/0.75	+	ERMR, ILMP, inverted ILM flaps insertion, C_3_F_8_	+; 20/125; 19
3	F/68	1–3/3/active	ND50 cm	FVPR, C_3_F_8_, ILMP	HM1m	Anterior PVR, CME	1065/1.25	+	Perfluorocarbon-assisted retinectomy, neurosensory retinal free flap insertion, SOI	+; 20/400; 12
4	M/65	1, 2/2/active	20/400	FVPR, C_3_F_8_^b^	20/1000	ERM	544/6.5	−	ERMR, ILMP, C_3_F_8_	+; 20/800; 9
5	F/54	2–4/3/active	HM20 cm	FVPR, C_3_F_8_, ILMP	20/200	ERM, LMH, CME	146/11	−	ERMR, ILMP, C_3_F_8_	+; 20/100; 6
6	M/58	2, 3/2/active	20/100	FVPR, SOI	HM60 cm	ERM, LMH	762/21	−	ERMR, ILMP, C_3_F_8_	+; ND20 cm; 6
7	M/53	1, 2/3/active	20/2000	FVPR	20/250	ERM, LMH, CME	209/76	−	ERMR, ILMP, temporal inverted ILM flap, C_3_F_8_^b^	+; 20/63; 14
8	F/60	1–4/4/active	HM1m	1^st^: FVPR, SOI; 2^nd^: SOE, retinectomy, eSB; 3^rd^: SOE, subfoveal fibrotic band removal^b^	ND10 cm	ERM, LMH, anterior PVR, chronic RD, CME	453/12	+	SOE, ERMR, ILMP, subfoveal band removal, laser supplement at MH margin	+; ND20 cm; 6
9	F/53	2, 3/3/active	20/200	1^st^: FVPR, SOI, eSB 2^nd^: SOR, FVPR	ND10 cm	ERM	446/36	−	ERMR, ILMP, temporal inverted ILM flap, C_3_F_8_	+; ND20 cm; 3

^a^Diagnosis: 1, VH; 2, FVP; 3, tractional RD; 4, rhegmatogenous RD; ^b^combined with cataract operation; ^c^performed at an outside hospital BCVA, best-corrected visual acuity; CME, cystoid macular edema; DV, diabetic vitrectomy; ERMR, epiretinal membrane removal; eSB, encircling scleral buckle; FVPR, fibrovascular proliferation removal; HM, hand motion; ILM, internal limiting membrane (ILMP, peeling); LMH, lamellar macular hole; MH, macular hole; ND, number of digits; Mos: months; PVR, proliferative vitreoretinopathy; RD, retinal detachment; VH, vitreous hemorrhage; SO, silicone oil (SOE, exchange; SOI, infusion; SOR, removal).

**Table 2:** Possible factors related to secondary macular holes formation after diabetic vitrectomy.

Etiology	Progression to MH (months)	Association with RD	Visual improvement	Frequency	Representative cases
Foveal thinning	0.63	50%	50%	22%	1, 2
Incomplete FVP removal	0.75	100%	100%	11%	2
Iatrogenic damage	0.75	67%	67%	33%	1, 2, 3
Persistent cystoid macular edema	6.1	50%	83%	67%	1, 2, 3, 5, 7, 8
Proliferative vitreoretinopathy	6.6	100%	100%	22%	3, 8
Epiretinal membrane	12.0	29%	57%	78%	2, 4, 5, 6, 7, 8, 9

FVP, fibrovascular proliferation; MH, macular hole; RD, retinal detachment.

## Data Availability

The data used to support the findings of this study are available on reasonable requests from the corresponding author.
